# The pig as a medical model for gynecological diseases: an applied perspective

**DOI:** 10.3389/fvets.2025.1668113

**Published:** 2025-12-17

**Authors:** Dan Zhao, Yanan Zhao, Beibei Zhang, Da Liu, Yongzhi Deng, Guangju Qi

**Affiliations:** 1 College of Exercise and Health, Jilin Sport University, Changchun, Jilin, China; 2 Institute of Acupuncture and Moxibustion, China Academy of Chinese Medical Sciences, Beijing, China; 3 Department of Rehabilitation I, Cangzhou Hospital of Integrated Traditional Chinese and Western Medicine, Cangzhou, Hebei, China; 4 School of Pharmacy, Changchun University of Chinese Medicine, Changchun, Jilin, China; 5 Department of Acupuncture, The Third Affiliated Clinical Hospital of Changchun University of Chinese Medicine, Changchun, Jilin, China; 6 Department of Women's Health, Tongde Hospital of Zhejiang Province, Hangzhou, Zhejiang, China

**Keywords:** pigs, gynecological diseases, large animal models, ovarian cancer, genetic engineering

## Abstract

Gynecological diseases pose substantial risks to female reproductive health and overall wellbeing. To improve disease prevention and treatment, researchers continue to develop experimental models that faithfully replicate key features of the female reproductive system. Porcine models have gained increasing attention in gynecological research due to their substantial similarities to humans in anatomical structure, physiological function, and pathological processes. This review aims to evaluate the practical applications of porcine models for investigating gynecological disorders. We examine the use of porcine models in studying common gynecological diseases such as endometritis, infertility, and ovarian cancer, analyze their advantages and limitations, and discuss their role in genetic engineering applications. Notably, porcine models have facilitated key advances in gynecological research, including elucidating neuropeptide-mediated uterine dysmotility in endometritis, refining surgical techniques for uterine transplantation and vaginoplasty in infertility management, and evaluating dihydroartemisinin as a potential therapeutic agent for ovarian cancer. This analysis aims to characterize the distinctive features of porcine models for gynecological research, thereby facilitating the selection of optimal animal models for future preclinical studies.

## 1 Introduction

Gynecological diseases represent a significant global public health challenge, adversely affecting women’s physical and mental well-being and contributing to substantial healthcare burdens (1, 2). Epidemiological studies underscore the scale of this issue; for instance, endometriosis affects approximately 10% of women of reproductive age, while infertility impacts about 8–12% of couples worldwide, with a significant proportion attributable to gynecological conditions such as ovulatory dysfunction and uterine fibroids (3, 4). The pathogenesis of these diseases is complex, often involving endocrine dysregulation, infections, and benign or malignant tumors, and they are frequently characterized by high recurrence rates (5, 6). This not only severely compromises women’s health but also imposes considerable social and economic costs (7).

A major obstacle in combating these diseases is the limited availability of effective treatments for many gynecological disorders. Research in this field critically depends on the use of animal models to study disease pathophysiology, evaluate drug safety and efficacy, and develop novel therapeutic strategies (8). An ideal preclinical model should accurately recapitulate human disease phenotypes and genotypes. While small animal models like rodents are commonly used due to their cost-effectiveness and ease of handling, they often fall short in replicating key anatomical, physiological, and genetic features of the human female reproductive system (9).

In contrast, pigs have emerged as a highly relevant large animal model for gynecological research. Compared to other large animal models—including sheep, rabbits, and non-human primates (NHPs)—pigs provide a more favorable balance of anatomical and physiological similarity to humans, practical feasibility, and ethical acceptability. Although sheep are valuable for certain reproductive studies, they differ significantly from humans in estrous cycle regulation and uterine anatomy (10, 11). Rabbits, while more manageable to house, show substantial differences in reproductive anatomy and lifespan relative to humans. Non-human primates, despite close phylogenetic relatedness, face limitations including high costs, prolonged reproductive cycles, and stringent ethical restrictions (12). In contrast, pigs share substantial reproductive anatomical (e.g., uterine structure, placental type), physiological (e.g., estrous cycle length, ovarian function), and genomic (~98% homology) similarities with humans. They also offer advantages in availability, shorter gestation periods, and fewer ethical concerns compared to NHPs (13). Their anatomical structures, physiological functions, hormonal regulation, and disease progression closely mirror those of humans (14). For example, the porcine uterus shares significant structural similarities with the human uterus, including a glandular endometrial layer and smooth muscle composition, which is vital for modeling conditions like endometriosis (15). Genetically, pigs and humans share approximately 98% of their gene sequences, facilitating transgenic studies and gene therapy research (16).

A systematic literature search was conducted in PubMed and Web of Science to ensure a comprehensive and reproducible review. Search terms comprised “porcine model,” “gynecological diseases,” “endometritis,” “infertility,” “ovarian cancer,” “genetic engineering,” and “transgenic pigs.” The search encompassed articles published from 2000 to 2025. The study selection process was based on screening titles and abstracts for relevance, supplemented by snowball sampling of references from key articles. The inclusion criteria were restricted to English-language publications, with a focus on original research and reviews concerning porcine models in gynecological disease research.

Therefore, this paper aims to provide a comprehensive overview of the current applications, advantages, challenges, and future potential of porcine models in gynecological disease research, with a view to recommending more suitable animal models for gynecological disease studies.

While previous reviews have discussed porcine models in general biomedical research, this work specifically highlights their unique applicability to gynecological diseases. It focuses on uterine transplantation, endometriosis, hormonal regulation, and genetic engineering—areas where porcine models provide distinct advantages over rodent and other species.

## 2 Pig and gynecological diseases

Porcine models have gained prominence in gynecological disease research due to advances in biotechnology. These models help elucidate disease mechanisms and provide a vital platform for developing and evaluating novel therapeutic strategies (17). Pigs share key similarities with humans in reproductive cycle, physiology, body size, and organ structure (18). Consequently, they are widely used to model female reproductive disorders including infertility and pregnancy complications, to evaluate surgical and interventional therapies, and to advance technologies such as xenotransplantation and gene editing (19, 20).

However, porcine models present several limitations, with a primary constraint being the substantial costs of breeding and maintaining large animals, as housing, feeding, and veterinary care requirements incur greater expenses than those for rodent models (21). Furthermore, despite anatomical and physiological similarities to humans, certain interspecies differences may limit how accurately porcine disease progression reflects human pathophysiology. For instance, although pigs share similar uterine anatomy with humans, their reproductive cycles and hormonal regulation differ, potentially altering responses to therapeutic interventions (22). These physiological disparities may constrain the complete applicability of porcine models for simulating human gynecological diseases.

**Figure 1:**
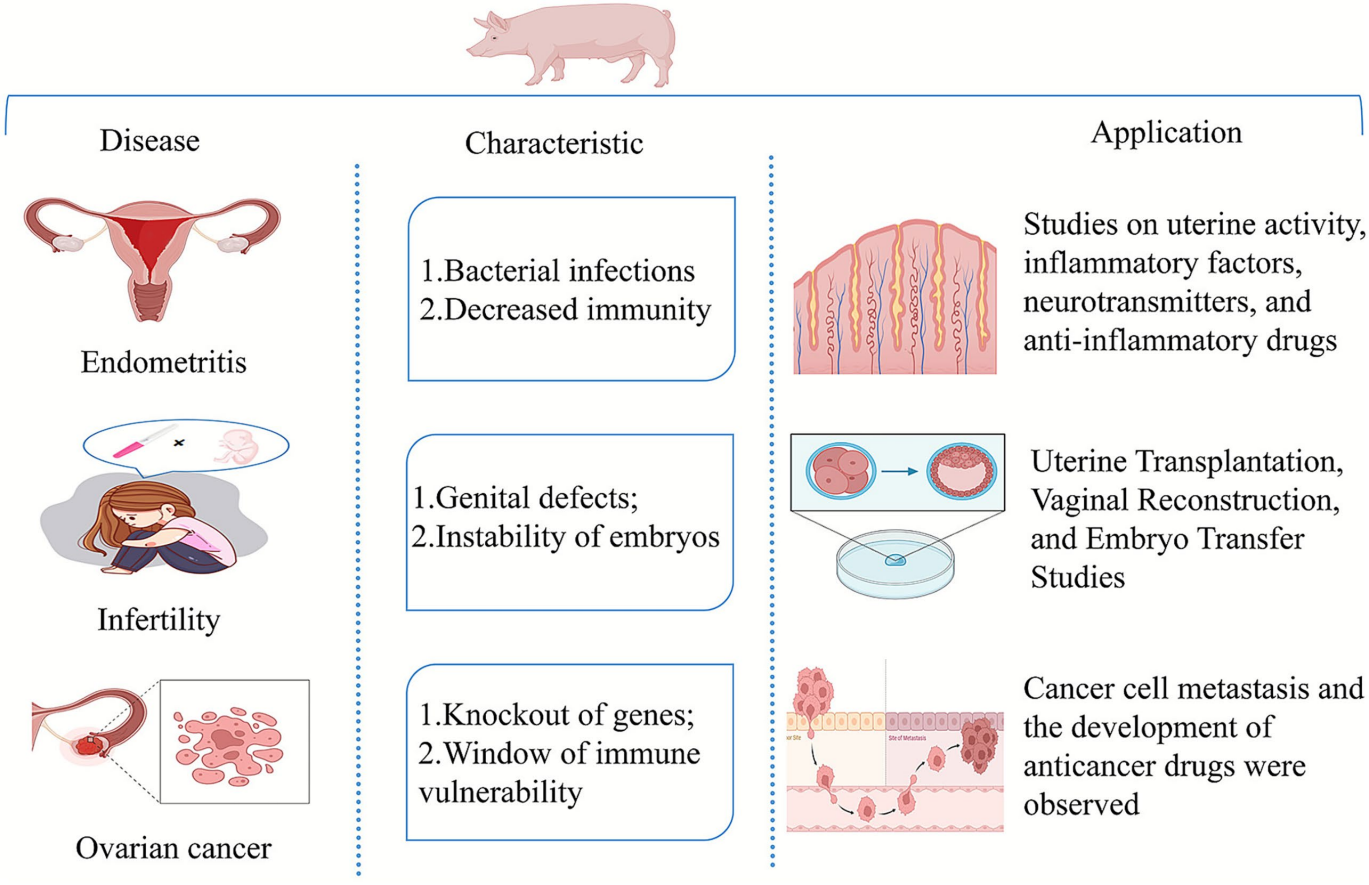
Application of pig model.

Despite these challenges, pigs have provided critical insights into various gynecological diseases. For instance, in ovarian cancer research, pigs have helped identify novel cancer treatment targets, and these findings have been successfully translated into clinical trials (23). Although direct references to human trials are not yet available for all targets, the mechanistic insights gained from porcine models support their continued investigation in translational oncology. Additionally, pigs have been used to explore the role of growth differentiation factor 9 (GDF9) in ovarian function, and insights into this molecular mechanism have informed infertility research strategies (24). Research into endometriosis in pigs has also contributed to the preclinical development of new drug therapies that are now undergoing clinical trials. Dihydroartemisinin (DHA) has shown significant effectiveness in reducing ovarian cancer tumor growth in pig models, and this preclinical evidence supports its potential for future clinical evaluation, demonstrating the potential of porcine models in cancer drug development (25). The applications of porcine models in studying prevalent gynecological disorders like endometritis, infertility, and ovarian cancer are illustrated in Figure 1.

### 2.1 Endometritis

The female reproductive tract—including the perineum, vulva, cervix, and uterine cavity—hosts diverse microbial communities (26). Multiple factors—including poor hygiene, prolonged labor, obstetric interventions, and retained placental fragments—can disrupt protective barriers at the vulvar lips, vestibule-vaginal junction, and cervix, consequently permitting ascending uterine infection and subsequent endometritis development (27). Endometritis, a common reproductive tract infection, is frequently caused by pathogens such as Streptococcus, *Escherichia coli*, and Staphylococcus (28). These pathogens disrupt the protective barriers of the female reproductive tract, enabling infection and subsequent endometritis development. In porcine models, endometritis is commonly induced via *E. coli* inoculation to mimic human infection. This infection induces mucinous or purulent inflammatory lesions on the endometrial surface, resulting in uterine inflammation and pain (29). To model endometritis in pigs, researchers typically inoculate bacterial pathogens such as *E. coli* into the uterine cavity. A standard induction method involves infusing *E. coli* suspension into porcine uterine horns, typically on the fifth day of estrus, rapidly producing inflammatory exudates marked by erythema, swelling, and vascular dilation of the endometrium. Researchers assess infection response through parameters including uterine contractility, inflammatory markers, and endometrial histology. Specific measurements include uterine contraction amplitude and frequency, along with cytokine levels such as TNF-*α* and IL-1 to quantify inflammatory responses. These markers elucidate inflammation progression and its functional impact on the uterus (30, 31). In advanced endometritis, persistent inflammation can significantly impair uterine contractility. Due to the lack of noticeable symptoms in early stages, untreated endometritis may progress to uterine cavity adhesions, leading to menstrual abnormalities and infertility that substantially affect quality of life (32). Recent studies demonstrate that myometrial function is regulated by myogenic, neurogenic, and hormonal mechanisms (33, 34). Uterine microbiota normalization can be promoted by regulating uterine contractions and immune responses (35).

Endometritis frequently affects pigs, usually resulting from bacterial infections following parturition stress or improper breeding management (36). High maintenance costs—including housing, feeding, and veterinary care—often restrict the number of animals feasible for research (37). Environmental factors such as temperature, humidity, and procedural stress can significantly influence experimental outcomes. Pigs are particularly stress-sensitive, and stress-induced immune modulation may confound experimental results (38). Although strict environmental controls are necessary to minimize these variables, such measures increase the complexity and expense of porcine studies. This condition reportedly accounts for 1.4 to 60% of overall disease prevalence in swine (39). Consequently, pigs are frequently selected as models to investigate factors regulating uterine function.

In porcine models of *E. coli*-induced endometritis, multiple neurotransmitters and neuropeptides—including norepinephrine, acetylcholine, calcitonin gene-related peptide, neuropeptide Y, vasoactive intestinal peptide, pituitary adenylate cyclase-activating peptide (PACAP), somatostatin, and galanin—modulate uterine contractility and inflammatory responses through their specific receptors (Table 1).

**Table 1:** Neurotransmitters and neuropeptides in porcine endometritis models.

Signaling molecule	Receptor(s)	Effect on uterine activity	Experimental context	References
Norepinephrine	α2-AR subtypes	Increases contraction	*E. coli*-induced endometritis; organ bath	(105, 106)
Acetylcholine	MR2, MR3	Decreases contraction	*E. coli* model; muscarinic antagonism	(107, 108)
CGRP	CGRPR	Promotes inflammation	Associated with exudate accumulation	(109)
NPY	Y1R, Y2R	Modulates contractility	Receptor expression changes in inflammation	(110)
VIP	VPAC1, VPAC2	Reduces uterine activity	Isolated from pig intestine; reduces tone	(111, 112)
PACAP	PAC1	Increases amplitude	Exogenous administration; receptor blockade	(40)
Somatostatin	Sstr5	Increases amplitude	Receptor-mediated myometrial enhancement	(113)
Galanin (GAL)	GALR1	Increases frequency	Enhances exudate clearance and motility	(41)

CGRP, Calcitonin Gene-Related Peptide; NPY, Neuropeptide Y; VIP, Vasoactive Intestinal Peptide; PACAP, Pituitary Adenylate Cyclase-Activating Polypeptide; GAL, Galanin; CGRPR, Calcitonin Gene-Related Peptide Receptor; Y1R, Y2R, Neuropeptide Y Receptor Type 1/2; VPAC1, VPAC2, Vasoactive Intestinal Peptide Receptor 1/2; PAC1, Pituitary Adenylate Cyclase-Activating Polypeptide Type 1 Receptor; Sstr5, Somatostatin Receptor Type 5; GALR1, Galanin Receptor Type 1.

These findings establish a neurochemical basis for uterine dysmotility during inflammation and support developing receptor-targeted therapies. For instance, PACAP enhances uterine contraction amplitude via PAC1 receptors in inflamed tissues, as confirmed through organ bath assays and receptor antagonism (40). Similarly, galanin promotes inflammatory exudate clearance and increases myometrial contraction frequency through GALR1 receptors (41).

Beyond neuropeptide pathways, lipopolysaccharide (LPS)-induced cellular models simulate inflammatory responses and identify regulatory microRNAs such as ssc-novel-miR-106-5p, which suppresses MAP3K14 expression and reduces pro-inflammatory cytokine production (42). Additionally, porcine models effectively evaluate the pharmacokinetics of anti-inflammatory drugs like carprofen and novel antimicrobials such as lysostaphin, which demonstrates prolonged intravaginal activity against Staphylococcal and Streptococcal strains relevant to human endometritis (43).

The *E. coli*-induced porcine endometritis model produces rapid and reproducible inflammatory responses, rendering it appropriate for acute intervention studies. In contrast, LPS-induced cellular models enable mechanistic analysis but lack the complexity of intact organ systems. The successful translation of lysostaphin and optimized carprofen formulations from porcine models to human applications validates their predictive value for pharmacokinetic and therapeutic development. These findings collectively highlight the value of porcine models for both elucidating pathogenic mechanisms and accelerating targeted endometritis therapy development.

### 2.2 Infertility

Infertility constitutes a major global health challenge affecting female reproductive health. Current estimates indicate infertility affects approximately 480,000 couples and 186 million individuals of reproductive age worldwide (44). Multiple factors contribute to female infertility, including genetic mutations, chromosomal abnormalities, ovulatory dysfunction, and uterine pathologies (45). Endometriosis—characterized by ectopic endometrial-like tissue—is a leading cause of infertility, affecting approximately 10% of reproductive-aged women. This condition causes pelvic pain and inflammatory processes that impair fertility (46). Organic infertility typically stems from congenital defects, medical conditions, and Asherman syndrome—a condition characterized by intrauterine adhesions (47). Young women often require hysterectomy due to gynecological conditions including uterine fibroids, endometriosis, and adenomyosis, which cause significant pain and discomfort (48, 49). Postpartum hemorrhage also represents a significant indication for hysterectomy (50). Female infertility presents substantial challenges for affected individuals and families, while also creating significant societal burdens (51). Accurate animal modeling of female infertility requires high physiological concordance with humans, demanding stringent criteria for reproductive function, hormonal regulation, and genetic factors. Due to their anatomical, physiological, immunological, and genomic similarities to humans, pigs are increasingly valued as preclinical models for infertility research, and consequently, researchers are increasingly utilizing porcine models to develop innovative therapeutic approaches.

Uterine transplantation techniques have recently emerged as a promising treatment for patients with absolute uterine factor infertility (52). Uterine viability in porcine models can be maintained for up to 17 h using a modified Krebs-Ringer bicarbonate buffer solution in an open perfusion system (53). Xenotransplantation studies using porcine models show promising outcomes for graft survival and functional recovery (54). Mayer-Rokitansky-Küster-Hauser syndrome, characterized by vaginal agenesis, disrupts normal menstrual function and can cause infertility (55). Porcine small intestinal submucosa consists of collagen-based extracellular matrix that supports the regeneration of host-native tissues (56). This biomaterial is widely applied in multiple medical fields, including body wall reconstruction, vascular grafting, and hernia repair (57). Incorporating porcine small intestinal submucosa into McIndoe vaginoplasty improves functional outcomes regarding vaginal dimensions, scarring, and dilation while reducing surgical complexity and donor site morbidity (58).

Porcine models have been utilized to investigate endometriosis, a disorder strongly associated with infertility. Anatomical and physiological similarities to humans—especially in pelvic anatomy and hormonal regulation—render porcine models appropriate for studying endometriosis-associated adhesions and inflammatory processes. These models enable evaluation of novel therapies designed to preserve fertility in affected women (59). Moreover, physiological parallels between porcine and human reproduction extend to assisted reproductive technologies (ART). Porcine models have contributed significantly to refining techniques including *in vitro* fertilization, embryo culture, and embryo transfer procedures. Porcine studies have optimized culture conditions and transfer protocols, directly informing human ART practices and enhancing success rates (60).

Postoperative injury constitutes a common cause of pregnancy-related complications (61). Postoperative peritoneal adhesions often compromise fertility by disrupting gamete and embryo transport through anatomical distortion of adnexal structures (62). Studies evaluating postoperative outcomes have established porcine models demonstrating adhesion incidence rates comparable to those in humans (63). Thus, porcine models facilitate comprehensive multi-organ assessment during laparoscopic procedures and enable histopathological evaluation of reproductive system risks following chemical exposure (64).

Infertility can also result from pregnancy loss due to environmental exposures, uterine abnormalities, and other causes (65). ART play a vital role in achieving successful pregnancy and term delivery (66). *In vitro* fertilization-embryo transfer represents the most established ART approach, with embryo transfer constituting a critical stage in this therapeutic process (67). Clinically, minimally invasive techniques are often preferred over surgical interventions (68). Laparoscopy not only reduces surgical trauma while enabling oocyte retrieval, embryo recovery, and monitoring of developmental stages during transfer, but its efficacy also varies with catheter selection, thereby influencing procedural outcomes (69). Porcine studies comparing laparoscopic catheters have identified 1.0 mm instruments as optimal for intra-abdominal manipulation, as they offer improved stability, tensile strength, and flexibility with minimal tissue trauma and can reliably transfer two to four embryos into the oviduct (70). Additionally, extracellular vesicles in uterine luminal fluid enhance maternal-fetal communication and support embryonic development (71). While human uterine fluid collection is challenging, pigs share relevant anatomical, physiological, and genomic similarities with humans. Porcine-derived extracellular vesicles regulate inflammatory responses and represent promising natural agents for optimizing the uterine environment (72).

Porcine models of uterine transplantation and vaginal reconstruction demonstrate superior anatomical fidelity and functional recovery relative to rodent models. For example, the 83% success rate of vascular anastomosis in porcine uterine autotransplantation closely parallels clinical outcomes in humans, underscoring the model’s translational relevance (73). Similarly, vaginoplasty utilizing porcine small intestinal submucosa exhibits superior biocompatibility and lower complication rates than procedures employing synthetic materials (74).

In summary, the porcine model serves as a uniquely valuable platform for infertility research, particularly for surgical interventions such as uterine transplantation and vaginoplasty, where its anatomical scale and physiological responses closely parallel human conditions. Although limitations including high maintenance costs and the need for optimized immunosuppression regimens persist, the model’s high translational fidelity in replicating complex reproductive procedures and assessing novel biomaterials confirms its essential role in advancing infertility therapeutics.

### 2.3 Ovarian cancer

Gynecological cancers—particularly cervical, ovarian, and endometrial malignancies—pose substantial challenges to women’s reproductive health (75). Ovarian cancer demonstrates the highest mortality rate among gynecological cancers (76). Ovarian cancer initiation and progression involve complex interactions between genetic alterations and microenvironmental factors (77). Due to limited human pathological data, animal models provide essential platforms for preclinical studies of cancer development, progression, and reproductive consequences.

To better mimic human ovarian cancer features, researchers transplanted primary ovarian cancer cells into immunodeficient pigs. The transplanted cells maintained morphological characteristics of the original human tumors in this SCID pig model (78). These findings establish pigs as valuable models for gynecological cancer research. Approximately 75% of ovarian cancer patients with solid tumors develop peritoneal metastases (79). Researchers leveraged the transient immunocompromised state of 4–5 week old piglets for experimental modeling (80). This approach, by introducing human cancer cells, enabled the successful establishment of ovarian cancer metastases and facilitates the evaluation of the efficacy and safety of intraperitoneal anticancer drugs for various solid tumors.

In anticancer drug development, dihydroartemisinin (DHA) has emerged as a promising candidate with potent antitumor properties. Studies using porcine ovarian cancer models demonstrate that DHA induces endoplasmic reticulum stress in granulosa cells and activates the PERK/eIF2α/ATF4 signaling pathway, ultimately affecting tumor cell viability, calcium homeostasis, apoptosis, and unfolded protein response signaling (81). Experimental evidence shows that DHA treatment activates this pathway and elevates expression of endoplasmic reticulum stress and apoptosis markers, including CHOP and caspase-3. These findings establish a mechanistic foundation for DHA’s antitumor efficacy and confirm the value of porcine models for studying stress-induced apoptosis in ovarian cancer. The porcine model system enables applications critical to ovarian cancer research, particularly through compatibility with clinical imaging modalities and surgical procedures (82). This compatibility facilitates long-term investigations of cancer progression and metastasis. Results from porcine studies have been validated in subsequent preclinical trials, providing compelling rationale for early-phase clinical evaluation of DHA in ovarian cancer therapy (83). Although human clinical trial data remain limited, porcine models have established a robust preclinical foundation supporting DHA’s antitumor potential.

Porcine models have significantly advanced diagnostic imaging and therapeutic development for ovarian cancer. Their substantial size and anatomical compatibility enable the application of clinical imaging systems—including MRI—for non-invasive tracking of tumor progression, metastasis, and treatment response (84, 85). For example, imaging studies in porcine models have elucidated drug distribution and tumor penetration profiles of intraperitoneal delivery systems—parameters challenging to evaluate in smaller animal models (86). Therapeutic trials in porcine ovarian cancer models—evaluating novel agents such as DHA or immunotherapies—leverage the capacity for repeated sampling, longitudinal imaging, and clinically relevant surgical procedures. These capabilities enhance understanding of pharmacokinetic and pharmacodynamic properties while improving the predictive value of preclinical data for human trials, thereby accelerating translation of diagnostic and therapeutic strategies to clinical practice.

Porcine ovarian cancer models, especially those employing SCID pigs and xenotransplantation, replicate human peritoneal metastasis and the tumor microenvironment more faithfully than rodent models (87). Utilizing piglets in metastasis studies capitalizes on their transient immunocompromised state, which facilitates robust engraftment and progression of human cancer cells (88). These models have proven invaluable for assessing intraperitoneal drug delivery systems and imaging techniques with direct clinical applications.

Porcine models effectively bridge a critical translational gap between rodent studies and human patients in ovarian cancer research. Their compatibility with complex surgical procedures and advanced imaging techniques, coupled with their capacity to support the growth and metastasis of human-derived tumors, establishes a robust and clinically relevant platform for therapeutic development. Future research should prioritize standardizing these models and expanding their application to investigations of the tumor microenvironment and immunotherapy response—areas where the porcine immune system provides distinct advantages.

Despite their substantial advantages, porcine ovarian cancer models have several limitations that must be considered. These limitations include the limited availability of well-characterized, species-specific cancer cell lines compared to the extensive libraries for rodents; a relative shortage of immunology-specific reagents for mechanistic studies in pigs; substantial costs and infrastructure requirements for maintaining large-animal cohorts; and a lack of universally standardized protocols for model generation and characterization. Addressing these constraints is essential for the appropriate use and further development of porcine models in oncology research.

## 3 Advantages and challenges of applications

The advantages of porcine models for gynecological disease research are well established, as summarized in Table 2. As a rapidly maturing mammalian species, pigs not only provide economic benefits and raise fewer ethical concerns than non-human primates, but their substantial body size and anatomical similarities to humans, including cardiovascular, digestive, and renal systems, also significantly enhance their utility in research (89). Notably, pigs share relevant features with humans in epithelial regeneration, skin architecture, subcutaneous fat composition, and post-injury endocrine metabolism (90). As omnivores with physiological similarities in tissue morphology, organ size, and metabolic characteristics, pigs are suitable for repeated sampling and complex surgical procedures (14). Pigs also exhibit high genomic and proteomic homology with humans, sharing nearly identical gene content (91). Consequently, porcine models enable the application of gene knockout, transgenic, and genetic engineering techniques for mechanistic investigation (92, 93). Nevertheless, recognized limitations necessitate continued refinement of experimental designs to enhance data validity and scientific rigor.

**Table 2:** Advantages and challenges of pigs in the study of common gynecological diseases.

Diseases	Experimental methods	Dominance	Challenge	Significance	References
Endometritis	*E. coli* induction	Acute endometritis has a higher incidence of modeling.	Single-strain induction cannot fully replicate the complexities of natural infection scenarios.	Widely used in experimental research.	(114–116)
	LPS induction	Species and cell specificity.	EECs at different stages of the reproductive cycle have different sensitivities to LPS.	New mechanism of bacterial-induced inflammatory response revealed.	(42, 117)
Infertility	Uterine transplant	1. Higher vascular anastomosis 2. Organ microstructure similar to humans.	Dosage of immunosuppressants difficult to detect.	Giving hope to absolute uterine infert.	(73, 118, 119)
	Vaginal remodeling	1. Meet the high demand for vaginal function, length, scarring, and dilatation; 2. Minimize the incision and reduce the difficulty of the surgical operation.	Submucosal grafts of the small intestine are more costly and prone to polyp growth.	Modified traditional McIndoe vaginoplasty.	(120–122)
	Embryo transfer	Minimally invasive and safe, facilitating observation and collection of oocytes and embryos.	Instrumentation needs further improvement.	Ensuring successful pregnancies to full-term delivery.	(123–125)
Ovarian cancer	Metastasize cancer cells	Piglets have a “window of vulnerability” for their immune system.	Inhibition of active synthesis of new antibodies in piglets.	For evaluating the efficacy and safety of intravenous or intraperitoneal anticancer drugs in various solid tumors.	(86, 126)
	DHA exposure	Increased apoptosis rate.	Toxic side effects are unknown.	Informing the use of DHA in women’s diseases.	(25, 127)

LPS, Lipopolysaccharide; EECs, Enteroendocrine cel; DHA, Dihydroartemisinin.

Beyond biological factors, porcine models pose substantial logistical challenges requiring careful consideration. Substantial housing space, specialized surgical and postoperative facilities, and the requirement for skilled large-animal personnel collectively increase the cost and complexity of porcine studies (21, 94). These logistical limitations can restrict the scale and accessibility of porcine research, especially for resource-constrained institutions.

The use of porcine models in biomedical research, particularly in gynecological studies, requires rigorous ethical review and strict adherence to animal welfare standards. All studies must implement the 3Rs principle: Replacement (preferring non-animal alternatives where feasible), Reduction (using the minimum number of animals necessary), and Refinement (optimizing procedures to reduce discomfort) (95). Surgical interventions, including uterine transplantation and cancer metastasis induction, require appropriate anesthesia, analgesia, and comprehensive postoperative care to ensure animal wellbeing (96). Additionally, genetic modification protocols require oversight by institutional animal care committees to ensure scientific justification for potential welfare impacts (97). Transparent documentation of ethical approvals and housing conditions is crucial for research credibility and reproducibility (98).

## 4 Emerging technologies and translational perspectives

### 4.1 Genetic engineering and porcine model development

**Figure 2:**
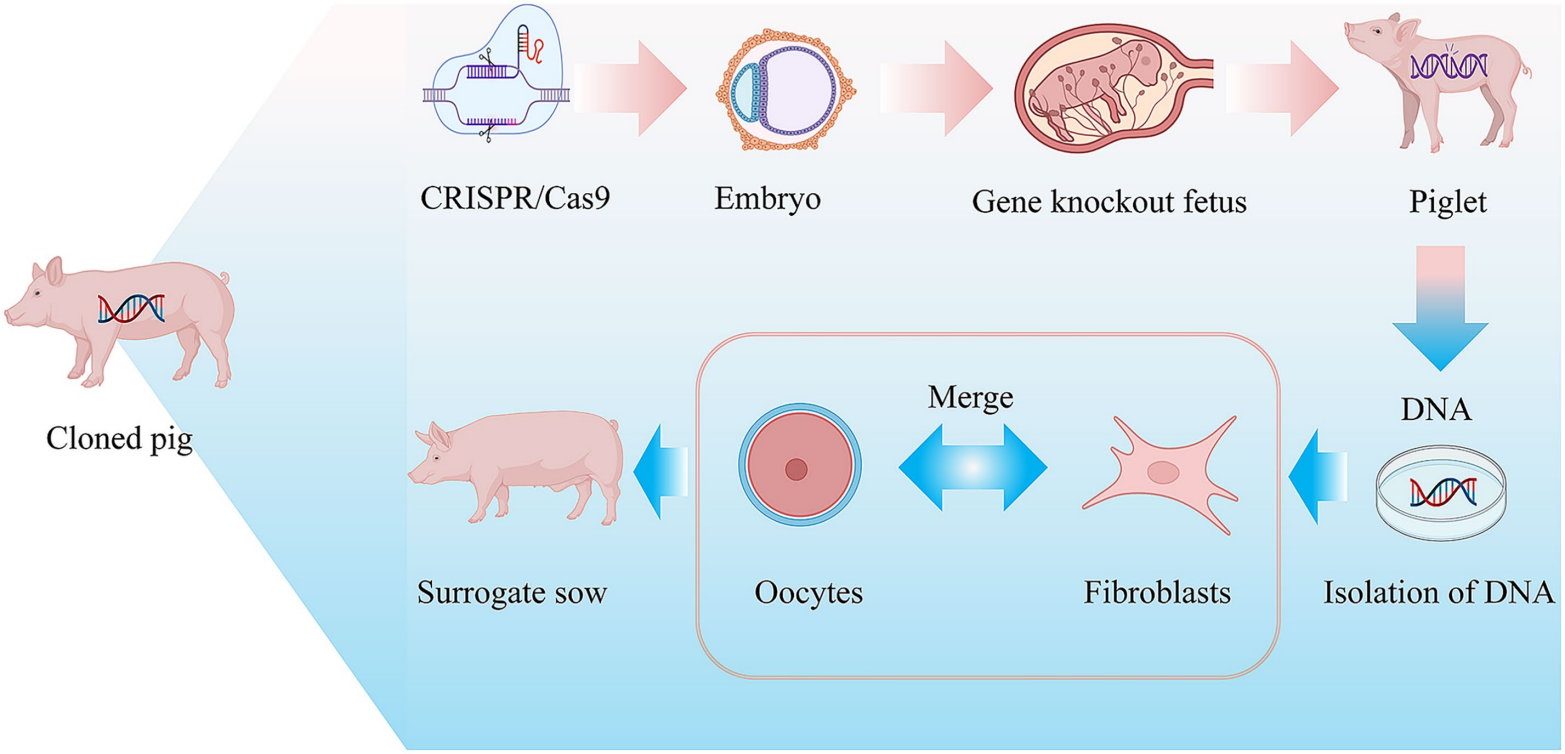
Application of pigs in genetic engineering.

Recent advances in genetic engineering, particularly CRISPR/Cas9 technology, have transformed the development of porcine models for gynecological research (see Figure 2). These technologies enable precise genetic modifications that recapitulate human disease phenotypes, ranging from single-gene disorders to complex cancer models (99, 100). The accelerated sexual maturation and substantial litter size of pigs facilitate efficient generation of these genetically defined models (101, 102).

### 4.2 Synergy between species characteristics and technological application

**Figure 3:**
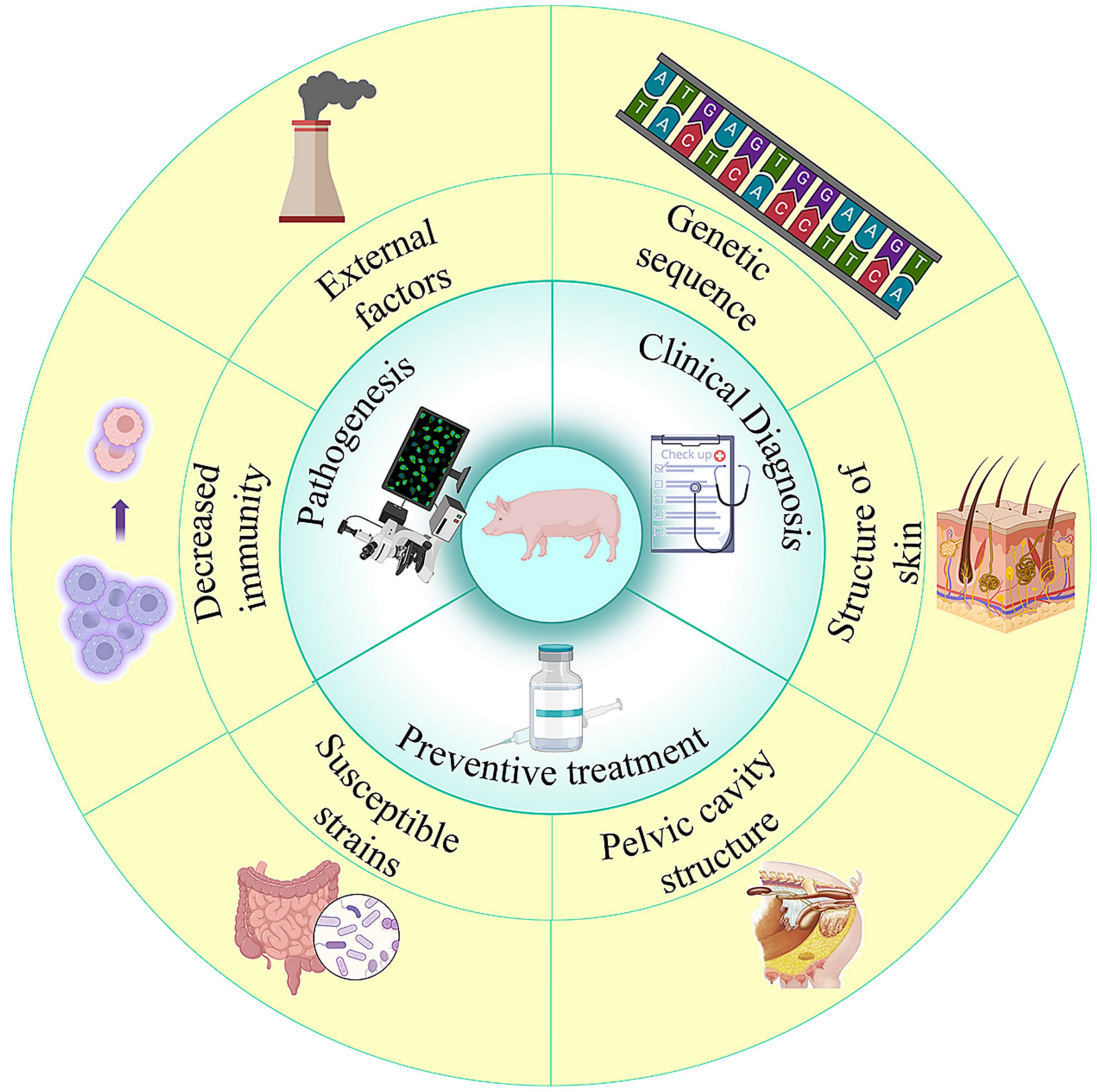
Application characteristics of pig species.

The success of genetic and surgical interventions in pigs stems from their substantial anatomical, physiological, and genomic similarities to humans (14). As summarized in Table 3 and Figure 3, the porcine reproductive system—including uterine anatomy, ovarian follicular dynamics, and estrous cycle—exhibits greater homology to humans than the rodent equivalent (103, 104). This biological congruence ensures that pathophysiological responses and therapeutic outcomes in porcine models show high predictive value for clinical translation. For example, the bicornuate uterus offers a distinctive anatomical environment for investigating tumor dissemination and local drug delivery, while similarities in hormonal regulation establish pigs as suitable models for studying polycystic ovary syndrome (PCOS) and endometriosis—areas of active model development (86).

**Table 3:** Differences in reproduction among humans, pigs and rats.

Species differences	Reproductive system	Estrus performance	Fertility characteristics	References
Female	Ovary size: 4 cm × 3 cm × 1 cm, Weight: 5-6 g	Spontaneous menstruation	Spontaneous ovulation	(104, 128)
	Pear-shaped uterus with mixed myometrium	Sexual maturity begins at age 18	Gestation period 259–294 days	(103, 129–131)
	The fallopian tubes are about 7–14 cm long and about 0.5–1.2 cm in diameter.	Menstrual cycle of about 28 days	1–2 babies/fetus	(128, 132, 133)
Pig	Ovary size: 5 cm × 3 cm × 2 cm, weight: 7-9 g	Spontaneous menstruation	Spontaneous ovulation	(104)
	Bicornuate uterus with a cylindrical uterine body and myometrium composed of longitudinal and circular muscle fibers	Sexual maturity begins at 8 months	Gestational period 114 days	(118, 134)
	The fallopian tubes are about 14–20 cm long and about 0.4–0.5 cm in diameter.	Oestrus cycle 18–23 days	10 babies/fetus	(13)
Rat	Ovary size: 0.2 cm × 0.1 cm × 0.05 cm, weight: 0.003 g	Menstruation is artificially induced.	Spontaneous ovulation	(104, 128)
	Bicornuate uterus, with a small uterine corpus, a distinct “Y” shape of the uterine horns or tubal connections, and a myometrium composed of longitudinal and rounded fibers.	Sexual maturity begins in the 4th week.	Gestational period 19–23 days	(130, 135, 136)
	Tubal length about 40-55 mm, diameter 0.3 mm.	The estrous cycle lasts about 4–5 days and it can come into estrous again after 12-24 h of parturition.	4–12 babies/fetus	(132, 137)

### 4.3 Future directions and integrated platforms

Future integration of porcine models with multi-omics approaches and advanced imaging technologies will significantly strengthen their translational potential. Standardized protocols for complex procedures such as uterine transplantation and long-term monitoring in genetically defined porcine lines are essential for generating reproducible, high-quality preclinical data. Continued refinement of these models, together with ethical and cost-effective husbandry practices, will consolidate the pig’s position as a crucial bridge between basic research and clinical applications in gynecology.

## 5 Summary

This review establishes porcine models as superior translational platforms for gynecological research. Their anatomical, physiological, and genetic homology to humans makes them particularly suited for studying complex gynecological conditions—including endometritis, infertility, and ovarian cancer—and for developing surgical techniques and therapeutics. Porcine models accurately replicate human uterine physiology and inflammatory responses, providing ideal systems for investigating endometritis pathogenesis and screening anti-inflammatory and antimicrobial agents. Additionally, demonstrated anatomical compatibility and functional recovery in uterine transplantation and biomaterial-based vaginoplasty models offer critical preclinical evidence for refining infertility interventions. In oncology, immunocompromised porcine models with xenografts reproduce key features of human ovarian cancer progression and peritoneal metastasis, creating robust platforms for evaluating intraperitoneal drug delivery and localized therapies. Advanced genetic tools like CRISPR/Cas9 further enable development of models that recapitulate specific disease mechanisms and assess emerging gene therapies.

Although challenges regarding cost, husbandry, and model standardization remain, continuous progress in genetic engineering and increasing recognition of their translational fidelity are progressively addressing these limitations. By capitalizing on the unique strengths of porcine models and overcoming current limitations, researchers can accelerate scientific discovery and improve the translation of promising results into clinical practice, thereby enhancing outcomes for women with gynecological diseases.

Future studies should prioritize developing refined porcine models for understudied conditions such as PCOS and endometriosis. Furthermore, establishing standardized protocols for complex interventions and long-term monitoring will be essential. These efforts are crucial to accelerate the translation of discoveries into clinical applications and improve outcomes for women with gynecological disorders.
